# Metagenomic Next-Generation Sequencing for Pathogenic Diagnosis and Antibiotic Management of Severe Community-Acquired Pneumonia in Immunocompromised Adults

**DOI:** 10.3389/fcimb.2021.661589

**Published:** 2021-06-01

**Authors:** Ting Sun, Xiaojing Wu, Ying Cai, Tianshu Zhai, Linna Huang, Yi Zhang, Qingyuan Zhan

**Affiliations:** ^1^ Capital Medical University China-Japan Friendship School of Clinical Medicine, Beijing, China; ^2^ Department of Pulmonary and Critical Care Medicine, Center of Respiratory Medicine, National Center for Respiratory Medicine, China–Japan Friendship Hospital, Beijing, China

**Keywords:** metagenomic next-generation sequencing, community-acquired pneumonia, immunocompromised host, antibiotic management, days of therapy

## Abstract

**Background:**

Metagenomic next-generation sequencing (mNGS) is a promising technique for pathogens diagnosis. However, application of mNGS in immunocompromised adults with severe community-acquired pneumonia (SCAP) is relatively limited.

**Methods:**

We retrospectively reviewed 23 immunocompromised and 21 immunocompetent SCAP patients with mNGS detection from April 2019 to December 2019. The performances of pathogenic diagnosis and subsequently antibiotic adjustment in immunocompromised SCAP patients were compared to immunocompetent SCAP patients. The defined by days of therapy (DOT) method was used for estimate daily antibiotic use.

**Results:**

There was a significant difference in the diagnostic positivity rate between mNGS and conventional test in both groups (*P*<0.001). Compared to immunocompetent patients, more mixed pathogens in immunocompromised patients were found (*P*=0.023). Before the availability of mNGS, the DOTs in immunocompromise patients were higher than immunocompetent patients (3.0 [3.0, 4.0] *vs.* 3.0 [2.0, 3.0], *P*=0.013). Compared to immunocompetent patients, immunocompromised patients had fewer full pathogen covered empirical antibiotic therapy (14.7% *vs*. 57.1%, *P*=0.022), more adjustments of antibiotic treatment (87.0%) *vs.* 57.1%, *P*=0.027). More than a half (13 of 23) SCAP patients in immunosuppressed group had reduced or downgraded antibiotic adjustments based on the results.

**Conclusions:**

mNGS may be a useful technique for detecting mixed pathogens and personalized antibiotic treatment in immunocompromised SCAP patients.

## Introduction

Severe community-acquired pneumonia (SCAP) is a common disease in intensive care unit (ICU) with a mortality rate of 30-50% (Montull et al., 2016; Cilloniz et al., 2018), which was higher in immunocompromising patients. Immunocompromising patients account for 20-30% of hospitalized CAP patients (Jain et al., 2015; Di Pasquale et al., 2019). The inability to obtain a precisely and timely pathogen diagnosis might lead to excessive antibiotic treatment and unnecessary healthcare costs. However, pathogenic diagnosis and adequate empirical treatment in immunocompromised patients is challenging because they are susceptible to common pathogens and numerous opportunistic pathogens (Ramirez et al., 2020). The low detection rate and time-consuming process for conventional pathogen tests (Jain et al., 2015) make these challenges bigger, and which is a key problem faced by clinicians.

In recent years, metagenomic next-generation sequencing (mNGS) has attracted much attention in infectious filed because of its high-throughput capacity and fast turnaround time (Mitchell and Simner, 2019; Xie et al., 2019; Zhou et al., 2019; Wu et al., 2020). For SCAP patients, the pathogen positivity rate was 90.3% for mNGS versus 39.5% for conventional methods (Wu et al., 2020). The feasibility of mNGS for bronchoalveolar lavage fluid (BALF), blood, sputum, transbronchial lung biopsy (TBLB) and even lung biopsy tissues (Li et al., 2018; Liu et al., 2019; Chen et al., 2020; Li et al., 2020; Wu et al., 2020) in patients with respiratory infections has been demonstrated in many literatures. However, the high cost affects the wide application of mNGS in CAP patients. Compared with the immunocompetent adults, the immunocompromised adults with SCAP are prone to a wide range of potential pathogens and need more individualized and targeted treatment. Then, we hypothesized that immunocompromised patients with SCAP may benefit more from mNGS.

In this study, we attempted to evaluate the value of pathogenic diagnosis and clinical antibiotic adjustment based on mNGS application in immunocompetent and immunocompromised adults with SCAP.

## Methods

### Study Participants

A retrospective analysis was conducted for SCAP cases with mNGS detection of BALF in our intensive care unit from April 2019 to December 2019. This study was approved by the institutional review board of the China-Japan friendship hospital. SCAP was defined in patients with either one major criterion or at least three minor criteria of the Infectious Diseases Society of America (IDSA)/American Thoracic Society (ATS) criteria (Metlay et al., 2019). Immunosuppression was defined according to the previous article (Ramirez et al., 2020): primary immune deficiency disease; active malignancy; receiving cancer chemotherapy; HIV infection with a CD4 T-lymphocyte count <200 cells/μL or percentage <14%; solid organ transplantation; hematopoietic stem cell transplantation; receiving corticosteroid therapy with a dose 20 mg prednisone or equivalent daily for ≧14 days or a cumulative dose >700 mg of prednisone; receiving biologic immune modulators; receiving disease-modifying anti-rheumatic or other immunosuppressive drugs. Patients were excluded if they had been discharged before mNGS results were obtained, if they combine with hospital acquired pneumonia (HAP) or if the acquisition time of BALF was more than 72 hours after ICU admission. This study was performed in accordance with the Helsinki Declaration of 1964 and its later amendments. Since the data were anonymous, the need for informed consent was waived.

### Microbiological Testing and Pathogenic Analysis

The BALF specimens were divided into aliquots and subjected to conventional microbiological test as well as mNGS test in a pairwise manner. Some specimens were sent to the Vision Medical Co., Ltd. (China) for the mNGS analysis, performing nucleic acid extraction, library construction, high-throughput sequencing, bioinformatics analysis, and pathogen data interpretation according to previous studies (Li et al., 2020; Zhang et al., 2020). The other specimens were sent to our microbiological laboratory using conventional methods for microbiological analysis, including bacterial/fungal smear and culture, *Pneumocystis jirovecii* (PC) smear (Grocott methenamine staining), acid-fast stain and real-time PCR including cytomegalovirus (CMV), influenza virus, respiratory syncytial virus, *Legionella, Mycoplasma* and *Chlamydia spp*.

The mNGS results were defined as infectious pathogens if one of the following criteria were met: (i) culture and mNGS identified same microbe, and the mNGS reads number was more than 50 from a single species (Li et al., 2018); (ii) Mycobacterium tuberculosis was considered positive when at least 1 read was mapped to either the species or genus level; (iii) Microbes solely identified by mNGS were considered pathogens if two of the following criteria were met: >30% relative abundance at the genus level in bacteria or fungi (Li et al., 2018); coverage rate of this microbe scored 10-fold greater than any other bacteria or 5-fold fungi higher than any other fungus (Miao et al., 2018).

### Clinical Data Collection and Antibiotic Treatment

Data were retrieved from the medical records, including demographics, laboratory test results, APACHE II score, SOFA score, CURB-65 score, and ICU special treatment data. Antibiotic treatment before ICU, initial antibiotic at ICU admission and adjustment later based on the pathogen results of mNGS were also been collected.

All patients underwent empirical antimicrobial treatment according to the Chinese Adult CAP Diagnosis and Treatment Guidelines and Official Clinical Practice Guidelines for Adults with CAP form the American Thoracic Society and Infectious Diseases Society of America (Cao et al., 2018; Metlay et al., 2019). Antibiotic regimens were adjusted later based on the results of microbiology tests combined with clinical condition, respiratory infection indicators and imaging. The days of therapy (DOT) was used to estimate daily antibiotic strategies (Valles et al., 2020). One DOT represented the administration of a single agent on a given day, regardless of the number of doses administered or dosage strength. A single patient receiving two antimicrobial drugs would be recorded as receiving 2 DOTs (1 for each drug administered) and so on according to the number of antimicrobials received daily.

### Statistical Analysis

The t-test was used to determine the normal distribution and uniformity of variance. The Wilcoxon rank test was used to calculate the variance of measured data that were not normally distributed or had variance homogeneity. Pearson chi-square test, Fisher exact test or the McNemar test were used for discrete variables where appropriate. Concordance between two test results was measured by the Free Marginal Kappa. The significance of agreement was considered as follows, based on Landis and Koch (1977): a kappa value of 0.8-1 indicating near perfect agreement; 0.6-0.8 indicating substantial agreement; 0.4–0.6 indicating moderate agreement; less than 0.4 indicated low agreement. Data analyses were performed using the SPSS 26.0 (IBM, Armonk, NY, USA) software. *P* values<0.05 were considered significant, and all tests were 2-tailed.

## Results

### Sample and Patient Characteristics

A total of 65 patients with SCAP were screened and 21 patients were excluded based on the exclusion criteria. 23 immunocompromised patients and 21 immunocompetent patients were included in the study. The demographic features and characteristic baselines in the current study were provided in **Table 1**. Before treatment, there were no significant differences in age, gender, CURB-65 and SOFA scores, OI (oxygenation index) and incidence of ventilator and septic shock, between both groups (P >0.05). The APACHE II score was higher in immunocompromised patients (P<0.001). Seven immunocompromised patients (30.4%) were active malignancy or received cancer chemotherapy, while sixteen patients (69.6%) received prolonged corticosteroid or immunosuppressive drugs therapy.

**Table 1:** Patient characteristics and baseline of two groups.

Characteristics	All patients (n=44)	Immunocompetent patients (n=21)	Immunocompromised patients (n=23)	*P* value
Age (yr)	64.0 (55.8, 69.2)	62.0 (55.0, 68.0)	65.0 (57.5, 70.5)	0.473
Male, n (%)	29 (65.9%)	15 (71.4%)	14 (60.9%)	0.460
HLOS before ICU (days)	3.0 (0.8, 6.2)	2.0 (1.0, 6.0)	4.0 (0.5, 8.0)	0.307
BALF acquisition time from admission (h)	46.5 (29.0, 54.5)	41.0 (24.5, 53.5)	48.0 (34.0, 58.0)	0.378
APACHE II score	14.0 (10.0, 18.0)	10.0 (9.0, 14.0)	15.0 (14.0, 18.5)	<0.001^*^
CURB-65 score	2.0 (1.0, 3.0)	2.0 (1.0, 2.0)	2.0 (1.0, 3.0)	0.629
SOFA score	4.0 (3.0, 7.0)	4.0 (3.0, 6.0)	4.0 (3.0, 7.0)	0.800
OI	137.0 (85.5, 186.5)	136.0 (81.1, 186.0)	145.0 (87.4, 179.0)	0.879
Mechanical ventilation, n (%)	17 (38.6%)	9 (42.9%)	8 (34.8%)	0.583
Septic shock, n (%)	12 (27.3%)	6 (28.6%)	6 (26.1%)	0.853
CRRT, n (%)	1 (2.3%)	0 (0.0%)	1 (4.3%)	1.000
ECMO, n (%)	10 (22.7%)	4 (18.2%)	6 (26.1%)	0.578
Lac (mmol/L)	1.5 (1.2, 2.2)	1.5 (1.2, 2.2)	1.6 (1.2, 2.1)	0.990
PCT (ug/L)	0.3 (0.1, 3.5)	1.1 (0.1, 4.9)	0.2 (0.1, 0.8)	0.098
WBC (10^9^/L)	8.0 (5.4, 11.5)	7.8 (6.8, 10.8)	8.2 (5.0, 14.2)	0.842
Hb (g/L)	115.0 ± 25.4	107.3 ± 26.8	107.3 ± 24.7	0.661
PLT (10^9^/L)	182.4 ± 87.7	196.7 ± 93.4	170.0 ± 82.6	0.450
Scr (mmol/L)	63.7 (50.3, 89.0)	61.3 (54.2, 67.7)	71.0 (49.4, 91.9)	0.632
ALT (IU/L)	30.0 (20.0, 54.0)	30.5 (20.8, 63.0)	27.0 (19.0, 48.5)	0.429
Alb (g/L)	31.4 ± 4.9	31.6 ± 4.5	31.3 ± 5.4	0.638
APTT (sec)	40.0 (35.7, 46.6)	41.1 (38.5, 49.0)	36.2 (35.3, 44.0)	0.125

*P < 0.05 was considered statistically significant. HLOS, hospital length of stay; ICU, intensive care Unit; APACHE, acute physiology and chronic health evaluation scoring system; SOFA, sequential organ failure assessment; OI, Oxygenation Index; Lac, Lactate; PCT, Procalcitonin; WBC, White blood cell; Hb, Hemoglobin; PLT, Platelet count; Scr, Serum creatinine; ALT, Alanine aminotransferase; Alb, Albumin; APTT, Activated partial thromboplastin time.

### Potential Implications of mNGS Pathogenic Diagnosis in Immunocompromised Patients

**Figure 1:**
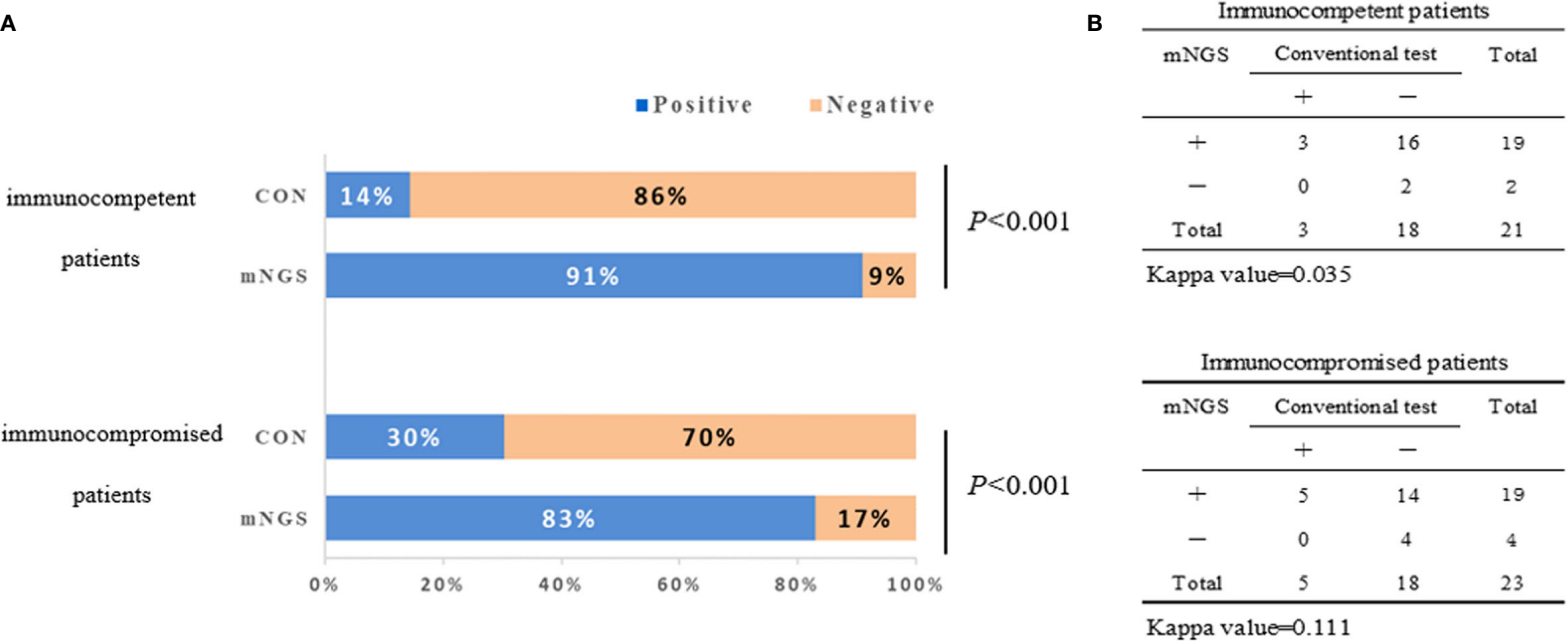
The positivity rate comparison and concordance analysis between metagenomic next-generation sequencing (mNGS) and conventional test. **(A)** The comparisons of positive rates (X-axis) for pairwised mNGS and conventional test in immunocompetent patients and immunocompromised patients (Y-axis) (*P*<0.001). **(B)** Concordance test showing there were low Kappa values of positive rates between mNGS and conventional test for both immunocompetent patients (0.035) and immunocompromised patients (0.111). mNGS, metagenomic next-generation sequencing; CON, conventional test.

**Figure 2:**
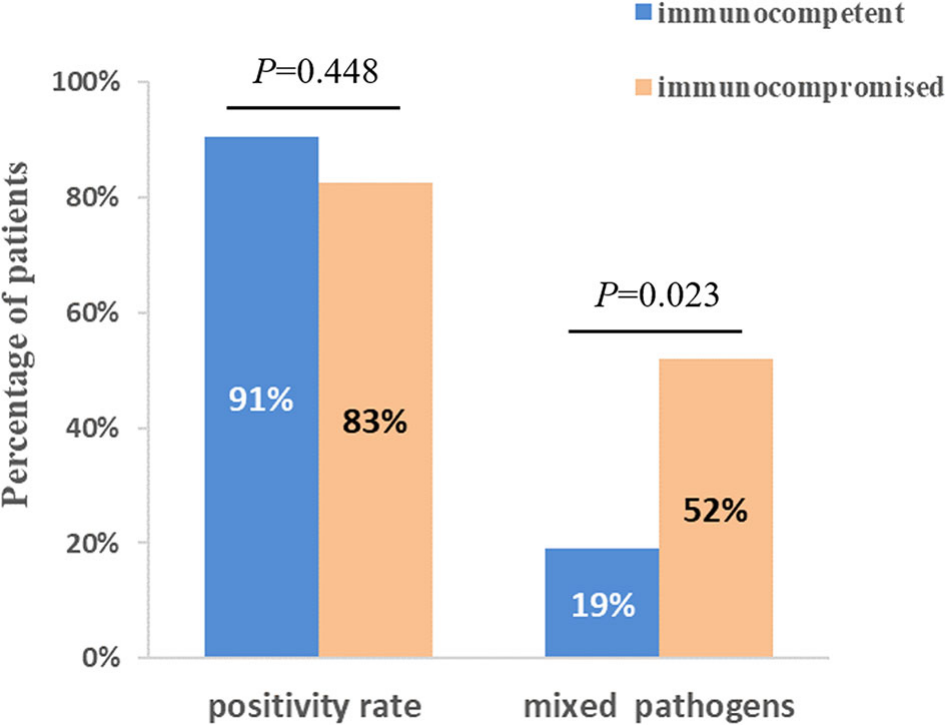
Comparison of positivity rate and detection of mix pathogens based on mNGS test between immunocompetent and immunocompromised patients. There was no significant difference between two groups when compared with positivity rate (*P*=0.448). 12 patients (52%) of immunocompromised patients were identified as mixed pathogen infection based on mNGS results, which were 4 (19%) in immunocompetent patients (*P*=0.023).

The diagnostic positivity rates of mNGS and culture test for both groups are illustrated in **Figure 1A**. There were significant differences in the diagnostic positivity rate between mNGS and conventional test in both groups (*P*<0.001; **Figure 1A**). Positive rates for immunocompetent patients and immunocompromised patients were 91% and 83% based on the mNGS test, but 14% and 30% based on the conventional test, respectively. There were low Kappa values in both groups (0.035 and 0.111, respectively; **Figure 1B**). There was no significant difference between the two groups in diagnostic positivity rate based on mNGS test (83% *vs.* 91%, *P*=0.448; **Figure 2**). Compared to immunocompetent patients, more mixed pathogens were found in immunocompromised patients (52% *vs.* 19%, *P*=0.023; **Figure 2**).

**Figure 3:**
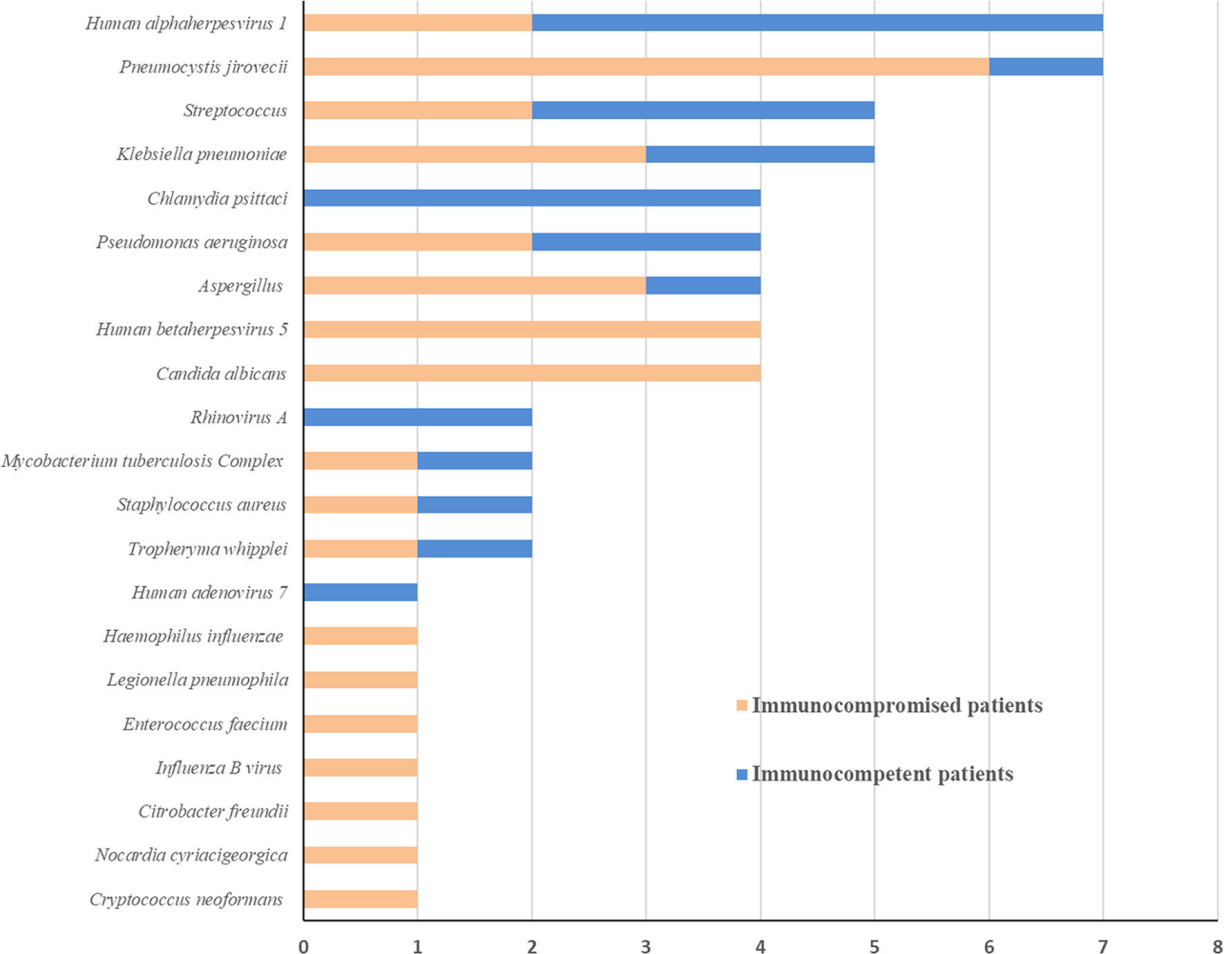
Pathogen spectrum of immunocompetent and immunocompromised patients with SCAP.

As shown in **Figure 3**, the spectrum of detected pathogens varied between immunocompetent and immunocompromised individuals. The most common pathogens were *Human alphaherpesvirus 1*, *Chlamydia psittaci*, and *Streptococcus* in immunocompetent patients, while *Pneumocystis jirovecii*, *Candida albicans*, and *Human betaherpesvirus 5*.

### Application of mNGS in Immunocompromised Patients for Antibiotic Adjustment

**Figure 4:**
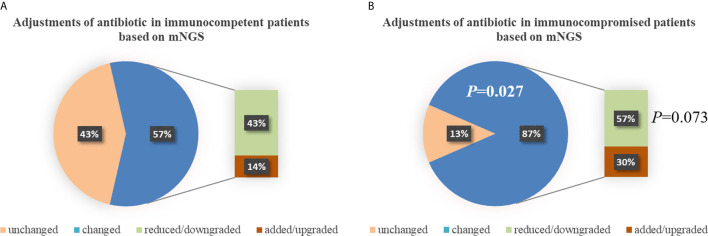
Adjustments of antibiotic in immunocompetent **(A)** and immunocompromised **(B)** patients. Compared to immunocompetent patients, immunocompromised patients had more antibiotic treatment adjustments (87% *vs.* 57%, *P*=0.027). There was a trend of reduced/downgraded antibiotic adjustments in immunocompromised patients compared to immunocompetent patients (57% *vs.* 43%, *P*=0.073), though there was no significant difference. mNGS, metagenomic next-generation sequencing.

There was no significant difference in the prior antibiotic exposure prior to ICU admission between both groups (76.2% *vs.* 73.9%, *P*=0.862; **Table 2**). Before the availability of mNGS, the DOTs were higher in immunocompromised than immunocompetent patients (3.0 [3.0, 4.0] *vs.* 3.0 [2.0, 3.0], *P*=0.013; **Table 2**). Compared to immunocompetent patients, immunocompromised patients had fewer full pathogen covered empirical antibiotic therapy (14.7% *vs.* 57.1%, *P*=0.022) and more antibiotic treatment adjustments (87% *vs.* 57%, *P*=0.027; **Table 2**). Although there was no significant difference, there was a trend of reduced/downgraded antibiotic adjustments in immunocompromised patients compared to immunocompetent patients (56.5% *vs.* 42.9%, *P*=0.073, **Figure 4**). More than a half (13 of 23) SCAP patients in immunosuppressed group had reduced or downgraded antibiotic adjustments based on the results. After the availability of mNGS, the DOTs were also higher for immunocompromised than immunocompetent patients (3.0 [2.5, 3.0] *vs.* 2.0 [2.0, 3.0], *P*=0.020; **Table 2**).

**Table 2:** The antibiotic treatment strategy before and after mNGS.

Treatment parameter	Immunocompetent patients	immunocompromised patients	*P* value
Prior antibiotic exposure, n (%)	16 (76.2%)	17 (73.9%)	0.862
DOTs of per patient before mNGS	3.0 (2.0, 3.0)	3.0 (3.0, 4.0)	0.013*
DOTs of per patient after mNGS	2.0 (2.0, 3.0)	3.0 (2.5, 3.0)	0.020*
Pathogen coverage of empirical antibiotic therapy based on mNGS			0.022*
Uncovered	1 (4.8%)	7 (30.4%)	
Partly Covered	7 (33.3%)	9 (39.1%)	
Covered	12 (57.1%)	4 (17.4%)	
mNGS was negative	1 (4.8%)	3 (13.0%)	
Do adjustment of antibiotic treatment based on mNGS pathogen	12 (57.1%)	20 (87.0%)	0.027*

*P<0.05 was considered statistically significant. DOT, days of therapy; mNGS, metagenomic next-generation sequencing.

## Discussion

This retrospective study compared the effectiveness of pathogen diagnosis and feasibility of antibiotic adjustment in immunocompromised patients with SCAP based on BALF mNGS. We have shown that mNGS may be a useful technique for detecting of mixed pathogens and personalized antibiotic treatment in immunocompromised SCAP patients.

This study compared the pathogenic diagnosis of immunocompetent patients and immunocompromised patients with SCAP. Firstly, the mNGS tests had higher positivity rate in both groups, compared with the conventional test, which is consistent with previous studies (Miao et al., 2018; Miller et al., 2019; Li et al., 2020). The Kappa values for concordance analysis between mNGS and conventional test were low in both immunocompetent patients and immunocompromised patients (0.288 and 0.125, respectively; a kappa value less than 0.4 indicated as low agreement). The lack of concordance was due to more detection and variety spectrums in the mNGS results. Zinter et al. (2019) found in BLAF from immunocompromised children, potential pathogens were detected in half of samples previously negative by clinical diagnostics. There was no significant difference between the positivity rates for both groups based on the mNGS test (*P*=0.448). Second, more mixed pathogens were found in immunocompromised than immunocompetent patients (*P*=0.023). In recent years, clinicians have become increasingly aware that the diagnosis of mixed pathogens is difficult in immunocompromised patients (Crotty et al., 2015). Consistent with our results, Wang et al. (2019) found that that mNGS had a higher sensitivity for mixed pulmonary pathogens detection than conventional tests. Third, the pathogen spectra of SCAP differed between immunocompetent and immunocompromised patients. In immunocompromised patients, *Pneumocystis jirovecii, Candida albicans*, and *Human betaherpesvirus 5* were the most detected pathogens. According to this data, the mNGS is a powerful method for microbiological diagnosis, especially for mixed infections in immunocompromised patients, which is consistent with previous reports (Parize et al., 2017; Wu et al., 2020). As such, mNGS may offers the probability for targeted therapy and improved clinical antibiotic overuse in immunocompromised patients.

A major innovative strength of this study was that to our knowledge, this is the first study to compare the antibiotic management of SCAP based on mNGS between immunocompetent and immunocompromised patients. Compared to immunocompetent patients, immunocompromised patients had more antibiotic treatment adjustments (87.0% *vs.* 57.1%, *P*=0.027), considering that there were no significant differences in baseline [*P*>0.05, except higher APACHE II score in immunocompromised patients because “immunocompromised” is an addition score (Knaus et al., 1985) in the APACHE II classification system] and prior antibiotic exposure rates (*P*=0.862). More mixed pathogen detections, higher DOTs of empiric and fewer full coverage may contribute to the more adjustments in antibiotic treatment with the availability of mNGS. It is also worth noting that, there were 13 (56.5%) of 23 immunocompromised patients had reduced or downgraded antibiotic adjustments based on the mNGS results, although there was no significant difference between both groups (*P*=0.073). Though the clinicians have gradually realized the particularity of immunocompromised SCAP patients, the optimized antibiotic strategy for them remains uncertain. The excessive antibiotic use can lead to resistance and medical resources waste (Webb et al., 2015; Metlay et al., 2019; Ramirez et al., 2020). Zhang et al. (2020) found in of immunosuppressed patients with acute respiratory distress syndrome (ARDS) caused by severe pneumonia, the detection of mNGS can significantly shorten the ICU stay, the ventilation time and reduce the ICU cost. The distinct advantage of NGS may can help clinicians more comprehensive evaluation of empiric antimicrobial therapy and make effective adjustments for immunocompromised SCAP patients.

Our research also has certain limitations. First, this study was limited by a small retrospective study. Second, most patients (33/44) had been hospitalised prior ICU, which may have affected the pathogens results. However, this is the real situation in our ICU. Third, considering the complexity of subsequently treament in immunocompromised SCAP patients, in this retrospective study, we only estimate the antimicrobial therapy before and after mNGS. To further investigation, we are conducting a prospective randomized controlled trial.

## Conclusion

In summary, we present an optimized mNGS that revealed advantages in pathogens diagnosis and implementation of targeted adjustment for empiric antimicrobial treament of immunocompromised SCAP patients. However, further investigations are required.

## Data Availability Statement

The data presented in the study are deposited in the NCBI repository. Accession numbers, SRA: SRP319801, BioProject: PRJNA729853.

## Ethics Statement

The studies involving human participants were reviewed and approved by Institutional review board of the China-Japan friendship hospital. Written informed consent for participation was not required for this study in accordance with the national legislation and the institutional requirements.

## Author Contributions

QZ conceived the study. TS, YC, TZ, LH, YZ, and XW performed data collection and analyses. TS prepared the first manuscript draft. QZ and XW provided major revisions and comments to the manuscript. All authors contributed to the article and approved the submitted version.

## Funding

This work was funded by National Key Research and Development Program of China (2016YFC1304300, QZ); Capital Clinical Features Applied Research and Achievement Promotion Project of Beijing, China (Z161100000516116, QZ); CAMS Innovation Fund for Medical Sciences (2018-I2M-1-003, QZ); Non-profit Central Research Institute Fund of CAMS (2019TX320006, QZ). Our research sponsors had no role in the research design, data collection, data analysis, data interpretation, or report writing.

## Conflict of Interest

The authors declare that the research was conducted in the absence of any commercial or financial relationships that could be construed as a potential conflict of interest.
